# A tonal-language benefit for pitch in normally-hearing and cochlear-implanted children

**DOI:** 10.1038/s41598-018-36393-1

**Published:** 2019-01-14

**Authors:** Mickael L. D. Deroche, Hui-Ping Lu, Aditya M. Kulkarni, Meredith Caldwell, Karen C. Barrett, Shu-Chen Peng, Charles J. Limb, Yung-Song Lin, Monita Chatterjee

**Affiliations:** 1 Centre for Research on Brain, Language and Music, McGill University, Rabinovitch House, 3640 rue de la Montagne, Montreal, Quebec H3G 2A8 Canada; 2 Chimei Medical Center, Taipei Medical University, Tainan, Taiwan; 3 Auditory Prostheses and Perception Laboratory, Boys Town National Research Hospital, 555 N 30th Street, Omaha, NE 68131 USA; 4 Department of Otolaryngology – Head and Neck Surgery, University of California San Francisco School of Medicine, San Francisco, CA 94115 USA; 5 Center for Devices and Radiological Health, United States Food and Drug Administration, Silver Spring, Maryland, USA

## Abstract

In tonal languages, voice pitch inflections change the meaning of words, such that the brain processes pitch not merely as an acoustic characterization of sound but as semantic information. In normally-hearing (NH) adults, this linguistic pressure on pitch appears to sharpen its neural encoding and can lead to perceptual benefits, depending on the task relevance, potentially generalizing outside of the speech domain. In children, however, linguistic systems are still malleable, meaning that their encoding of voice pitch information might not receive as much neural specialization but might generalize more easily to ecologically irrelevant pitch contours. This would seem particularly true for early-deafened children wearing a cochlear implant (CI), who must exhibit great adaptability to unfamiliar sounds as their sense of pitch is severely degraded. Here, we provide the first demonstration of a tonal language benefit in dynamic pitch sensitivity among NH children (using both a sweep discrimination and labelling task) which extends partially to children with CI (i.e., in the labelling task only). Strong age effects suggest that sensitivity to pitch contours reaches adult-like levels early in tonal language speakers (possibly before 6 years of age) but continues to develop in non-tonal language speakers well into the teenage years. Overall, we conclude that language-dependent neuroplasticity can enhance behavioral sensitivity to dynamic pitch, even in extreme cases of auditory degradation, but it is most easily observable early in life.

## Introduction

### Tonal language benefit in pitch

Speakers of a tonal language are continuously exposed to inflections in pitch, both rapid inflections within syllables to signify lexical tones^1,2^ and slower inflections at the sentence level, to communicate prosody^3^. This places a strong informational emphasis on pitch which appears to influence its neural coding, both in the brainstem and cortex. For example, at the cortical level, pitch processing generally activates the right hemisphere, but could engage the left hemisphere when the pitch contours are linguistically relevant as in tonal languages^4–6^. Frequency following responses (FFRs) recorded at the brainstem can also reflect pitch processing^7^, and this is where researchers have sought evidence for a more robust encoding of periodicity in speakers of tonal language. A large body of work by Krishnan and his colleagues has repeatedly shown that while FFRs preserve pitch information of lexical tones in both tonal and non-tonal language speakers, they are more robust in the former (see review^8^).

The question arises as to how generalizable this enhancement in pitch coding is in speakers of tonal languages, e.g. how sensitive it is to the particular curvatures of pitch contours inherent to a given language. A first study^9^ found no differences in pitch strength and pitch tracking accuracy derived from FFRs recorded in Mandarin and English speakers in response to linear fundamental frequency (F0) sweeps. Using iterated rippled noises, a second study^10^ recorded FFRs in response to four stimuli: a prototypical and three artificial contours (an inverted version of tone 2, a linearly rising sweep, and a tri-linear approximation of tone 2). They found that Mandarin speakers exhibited higher pitch strength than English speakers only with the prototypical contour; population differences were lost for the three artificial contours. Also using iterated rippled noises, a third study^11^ showed that Mandarin speakers showed larger mismatch negativity responses (a cortical event-related response giving a look at sub-cortical, pre-attentive, processing) than English speakers to curvilinear pitch contours modelled after Chinese tones, but this population difference was lost when using linearly rising sweeps. Thus, these three studies arrived at the conclusion that the sharpening of pre-attentive processing of F0 is specific to contours that are ecologically relevant. A later study^12^ tempered this view. On one hand, pitch tracking accuracy and pitch strength (similarly derived from brainstem FFRs in response to iterated rippled noises) were larger in Chinese than in English speakers, for sections of a lexical tone with rapid F0 changes, reinforcing the specificity of the tonal language advantage to particular curvatures in dynamic pitch contours. On the other hand, the effect was also observed with static F0 changes spanning a major third interval^12^. This musical interval was chosen to match the onset and offset of the lexical tone, and this is perhaps why the effect transferred to some degree outside of ecologically relevant stimuli (in Mandarin). Overall, it seems fair to conclude that, within neurophysiological studies looking at FFRs, the neural enhancement of pitch coding exhibited by speakers of tonal languages is most easily observable with ecologically relevant stimuli.

Another point of debate is the extent to which this neural enhancement translates into behavior. One of the earliest studies on this topic^13^ already described the inconsistent status of the literature at the time on this question, and failed to find the hypothesized tonal language benefit in pure tone discrimination (in fact, observing the opposite, a tonal language deficit in pitch sensitivity). Another study^14^ found no difference between Mandarin and English speakers in their ability to discriminate pure tones or pulse trains (while Mandarin speakers outperformed English speakers in Mandarin tone identification, as expected). In one of the FFR studies^15^, the neurophysiological enhancement did not translate into a perceptual advantage between Chinese and English speakers, and no correlation between F0 difference limens and FFR F0 magnitude was observed for these two groups. In contrast, two studies reported a tonal language benefit for pure tones in static discrimination or interval discrimination^16,17^. Using a very large sample through on-line testing, a tonal language benefit was revealed in detection of out-of-key incongruity^18^. Finally, a comprehensive study^19^ reported an advantage for Cantonese over English speakers in a number of perceptual tasks (pitch discrimination, pitch speed, pitch memory, and melody discrimination). As pointed out by Bidelman and his colleagues^19^, differences in experimental tasks/designs are likely to account for some of these apparent discrepancies. First, the pitch of pure tones, pulse trains, or iterated rippled noises, may not be directly relevant to voice pitch or musical pitch. Second, some studies may not have tested sufficiently small frequency differences^16^, have included listeners from multiple language backgrounds^16,17^ or have little audiological control^18^. Third, the cognitive abilities of participants could matter greatly in this phenomenon. For example Cantonese speakers exhibited superior working memory capacity (similar to English-speaking musicians) relative to non-musically trained English speakers^19^. This is a factor that could be particularly problematic with smaller-sample studies. One factor that has not been considered in previous studies is the developmental trajectory of brain plasticity. Given that younger brains are more plastic and yet also still developing cognitive skills, the tonal language advantage for pitch sensitivity may be more observable among children than among adults. Adults and older adolescents are likely to compensate for a lack of the tone-language advantage with more advanced cognitive skills and greater experience with diverse auditory inputs.

### Cochlear implant drawback for pitch

Cochlear implants (CI) are devices implanted surgically that allow people with profound hearing loss to recover hearing to some degree. Despite stringent limitations in spectral resolution - among other aspects of signal degradation - CIs generally provide enough auditory information for speech to be intelligible, as long as the background environment is relatively quiet^20^. They achieve this feat by delivering electrical pulses to the cochlea that are modulated as a function of the acoustic input captured by an external microphone. Envelope-based coding strategies attempt to reproduce the modulations of temporal envelopes extracted from different frequency bands of the acoustic signal, thus recovering a sense of articulation that is critical to intelligibility^21,22^. Such strategies were designed for speech perception, but not for the complex harmonic pitch of the human voice or of musical instruments. This does not mean that pitch is impossible to perceive through CIs but rather, that it has to be retrieved by cues that are largely sub-optimal, resulting in a percept that is not as salient as in normal hearing^23–25^.

At first sight, it may seem counter-intuitive to study the possibility of a tonal language advantage among CI users given their limitations in pitch perception. But CI users have proven very useful to our understanding of normal hearing, much in the same way as investigating disease improves our understanding of health. More precisely, the common interpretation of the tonal language advantage is that it sharpens and strengthens the fine-grained representation of periodicity (as reflected by FFRs analysis). Most CI users (using envelope-based coding strategies) lack this fine-grained representation, and therefore there should not be any tonal language advantage within this population for pitch coding per se. If we were to find such an effect in CI users, this would imply an alternative mechanism for this phenomenon.

### Goal of the present study

All studies aforementioned (section A) used adults only. To our knowledge, there is currently no data on the developmental trajectories of the hypothesized advantage (for pitch perception) of speaking a tonal language. As the phenomenon is directly related to language experience, it is a reflection of sensory neuroplasticity. As neuroplasticity changes dramatically in childhood, it is crucial to examine the effect across the developmental years. Thus, the main question addressed in the present study is the extent to which the tonal language effect holds within pediatric populations. In principle, younger brains should be more plastic. Thus, any neural enhancement in pitch coding should be more transferable to ecologically irrelevant pitch contours in developing children, while adults’ brains might be more sharply tuned to the exact curvatures of pitch contours occurring within the tonal environment. From this standpoint, one may hypothesize that the tonal language benefit would be *more easily observable* in children than in adults. One reason this might not happen is if the developing brain of a child speaking a tonal language had not fully specialized yet to process lexical tones as efficiently as adults, but this seems unlikely since typically developing children acquire mastery of lexical tones very early, before five years of age^26,27^. In the case for children with CIs, their brain is certainly not as specialized as their NH peers to process lexical tones^28,29^, but it has to adapt to a larger range of artificial/distorted sounds compared to NH children. So, in this sense, neuroplasticity may be even more important for children with CIs than for children with NH.

A second factor that may play a role is cognitive development. Adults may be able to compensate for their brain rigidity with their more advanced cognitive abilities. Thus, adult speakers of tonal and non-tonal languages may perform a pitch processing task using different mechanisms and skills than children of tonal and non-tonal languages. As in adults (previous studies in section A), we would expect to see minimal differences in our pitch sensitivity tasks between older adolescents who speak tonal or non-tonal languages, and we hypothesize that the difference would be greater for the younger children.

In a previous study^30^ involving more than a hundred children, we investigated this very question and found no difference in F0 discrimination abilities between NH Taiwanese and NH Americans or between Taiwanese CI users and Americans CI users, but a large deficit was exhibited by the CI populations in both countries. This result provided no support for the tonal-language plasticity hypothesis in children. We reasoned that our static F0 discrimination task did not tap into the neural stages that were hypothetically sharpened by tonal language environments, because (1) broadband complex tones with static F0 such as those used in our studies are too remote from ecologically-relevant curvilinear tones, and (2) the effect could be highly task-dependent (present in an identification or labeling task; absent in a discrimination task, as noticed earlier^14,31^). Here, we revisited the hypothesis of a tonal language benefit in a behavioral study, using both a labelling and a discrimination task focused on dynamic F0 processing. In an earlier report that included only English-speaking listeners^32^, we used 300-ms long broadband harmonic complex sweeps with a range of linear slopes from very-shallow to very-steep. As expected, we found substantial deficits in F0-sweep sensitivity by children and adults wearing CIs compared to their NH peers. Interestingly, we also found age-related differences: adults outperforming children, and older children outperforming younger children. However, these differences were largely common to NH and CI subjects, suggesting that the role of cognitive development might not interact substantially with hearing status in this task. We now turn to the role of language-dependent plasticity, which might vary across age and hearing status.

We address the following critical questions: can a tonal language environment sharpen sensitivity to linearly rising/falling F0 in children with NH? If so, at what age? And does this benefit occur in their peers with CIs? Differences between the two tasks should reveal something about the nature of the hypothesized benefit. If speaking a tonal language acted as an enhancer of the internal representation of F0 (e.g. as reflected by the FFR F0 magnitude), it should provide benefits in both the labelling and the discrimination tasks. In contrast, if speaking a tonal language acted more at an abstract level (e.g. extraction of coarse features that are linguistically relevant), it could provide a specific advantage in the labelling task. This seems plausible since Mandarin-speaking listeners may naturally process pitch in a dynamic context - for instance, as rising versus falling - and hence may find the present tasks more intuitive than English-speaking listeners who generally define pitch on a scale going from low to high in a musical context. English speakers would encounter dynamic pitch in speech in the context of prosodic cues, but those are generally slower, occurring over the course of a sentence, and perhaps less crucial to comprehending the meaning of utterances than lexical tones, which are an integral component of words in Mandarin.

## General Methods

### Subjects

Four groups of children participated. They included 44 Americans with NH (21 of whom had previously been reported^32^), 53 Taiwanese with NH, 52 Americans with CI (23 of whom had previously been reported^32^), and 45 Taiwanese with CI. The chronological age of all participants varied from 6.1 to 19.5 years. Details for each group are provided in Table 1. A large majority of the children with CI (45 Americans and all Taiwanese) were profoundly deaf at birth or within their first year of life. Age at implantation varied from 4 months to 14 years. Their duration of CI experience varied from 6 months at minimum up to 16.6 years. A minority of Americans (10) were unilaterally implanted (3 on the left side, 7 on the right), while 45 were implanted on both sides. In contrast, a majority of Taiwanese (39) were unilaterally implanted (16 on the left side, 23 on the right), while only 6 of them were implanted on both sides. Among the children implanted unilaterally, about half of them had sufficient residual hearing to wear a hearing aid on the contralateral ear. All children with CI, however, were tested on one ear only. Children with two implants were asked to unplug the most recent implant. For children with a single implant, ear-foam plugged the contralateral ear and any hearing aid was removed.

**Table 1:** Demographics of the four groups of children.

	Chronological age mean (std.) [min – max]	Age at implantation mean (std.) [min – max]	Duration of CI experience mean (std.) [min – max]	Age at profound hearing loss mean (std.) [min – max]
NH – US (n = 44)	11.0 (2.8) [6.1–18.1]			
NH – Taiwan (n = 53)	11.0 (2.9) [6.9–16.8]			
CI – US (n = 52)	12.5 (3.3) [7.8–19.5]	2.8 (2.8) [0.3–14.0]	9.7 (3.7) [0.5–16.6]	0.7 (2.0) [0.0–12.0]
CI – Taiwan (n = 45)	10.5 (3.3) [6.6–17.2]	2.9 (1.8) [1.0–12.2]	7.6 (3.4) [1.2–15.2]	1.0 (0.7) [0.0–2.3]

A large majority of Taiwanese (39) had a *Cochlear* device. Only two children had an *Advanced Bionics* device, and four had a *Med-El* device. American children were roughly split between *Cochlear* (24) and *Advanced Bionics* (26) devices, with only two children wearing a *Med-El*. All stimulation strategies were envelope-based (mostly ACE strategy for Nucleus 24, N5 and N6; mostly HiRes strategy for the Clarion, Naida, or Neptune; and the standard strategy for the Sonata and Opus2). Implanted children did not switch to a music program in their processor for this study: they used their CI as it was clinically assigned to them on a daily basis.

### Stimuli

The stimuli were identical to those presented in the earlier report^32^. Harmonic complexes were generated with all their partials up to the Nyquist frequency, with equal amplitude and in sine phase. The flat spectral envelope of these stimuli leaves little room for NH listeners to focus on individual harmonics within a particular frequency region. Instead, listeners must derive a dominant pitch percept by integrating periodicity information across spectral channels. For CI users, the same cannot be said with any degree of certainty: some subjects could be sensitive to a particular region of the cochlea with a better electrode-neuron interface or with higher number of healthy neurons to relay the auditory information higher up. Different processing strategies (variable across devices and manufacturers) could also promote listening to certain channels. Thus, it remains unclear how individual CI users derive their percept of global pitch from these stimuli (whether it is from envelope periodicities, or spectral centroid, or an even rougher comparison of place pitch across adjacent electrodes). However, for both NH and CI listeners, the percept of spectral edge pitch was eliminated by low-pass filtering at 10 kHz using Butterworth sixth-order filter with a slope of −30 dB per octave. The duration of the complexes was fixed at 300 ms to retain relevance to syllabic durations in speech, gated with ramps of 30 ms. The interval between stimuli was also set at 300 ms. In order to obtain a view of the entire psychometric function for every subject, sweeps had to range from very shallow (for NH subjects) to very steep (for CI subjects). Thus, the F0 of the complex could vary in a logarithmic space with rates of ±0.5, 1, 2, 4, 8, 16, 32, 64, and 128 semitones per second, and the scale was adjusted for individual subjects for performance to vary from chance to ceiling. Sweeps with opposite direction always shared the same F0 range, such that judgements had to rely on the evolution of the pitch percept over time rather than the overall pitch range that was covered. This was critical because CI users were expected to perceive many of the sweeps as flat, and a natural consequence is to start listening to the height of the pitch elicited rather than where it is heading towards. In the same logic, the base F0 was roved across trials between 100 and 150 Hz, to discourage children from relying on F0 range. All stimuli were generated at 65 dB SPL and presented with ±3 dB level roving.

### Protocol

After explaining the protocol and obtaining informed written consent from children and parents, the participants were invited to sit in the auditory booth and practice blocks commenced. The first task was a forced-choice procedure with a single interval and two alternatives (1I-2AFC). Children listened to a single F0-sweep and reported whether the pitch was rising or falling. The second task was a forced-choice procedure with three intervals and two alternatives (3I-2AFC). One F0-sweep was played as a reference, followed by two others, either identical or in opposite direction (randomly placed) at the same rate. Children reported the interval that sounded *different* from the reference. Before testing took place, practice blocks were presented without level roving, and with 20 trials to evaluate performance with the most extreme sweeps, namely 128 semitones/sec. The test was only initiated provided that children obtained at least 80% correct, averaged over the two directions (up or down). A test block usually contained 140 trials (7 rates by 2 directions, tested 10 times each, all shuffled). The seven rates ranged from 0.5 to 32 semitones/sec for NH listeners, and ranged from 2 to 128 semitones/sec for CI listeners. Once this first block was completed, the experimenter looked at the data collected and adjusted the scale of sweep rates for subsequent blocks if performance was too close to floor or ceiling, such that enough data could be obtained in the middle of the psychometric function.

The interface consisted of animated cartoons synchronized with presentation of the sounds and buttons on the screen^33^. In each trial, reaction time (RT) was recorded, although subjects were not aware of it. Feedback was provided via smileys and the experimenter tried to keep each child motivated by challenging him/her to win points and bonuses (not used for analysis). Experimental sessions lasted about one hour with short breaks between blocks. All children were paid for their participation. This study was approved by the Institutional Review Board of Johns Hopkins, BTNRH, UCSF, Chi Mei Medical Center and the Chang Gung Memorial Hospital, in accordance with principles expressed in the Declaration of Helsinki.

### Equipment and testing sites

The study took place at five research facilities. Taiwanese data were collected at the Chi Mei Medical Center in Tainan (52%) and at the Chang Gung Memorial Hospital in Taoyuan (48%). American data were collected at Boys Town National Research Hospital in Omaha (48%), and at the School of Medicine of UCSF in San Francisco (30%), and at Johns Hopkins Hospital in Baltimore (22%). The effect of experimental site in the US as well as in Taiwan was tested in each task but never reach significance (p > 0.153). The setups were broadly similar across sites. Stimuli were sampled at 44.1 kHz with a resolution of 16 bits. They were presented via a loudspeaker placed half a meter from the subject at 65 dB SPL. The loudspeaker (SB-1 Audio Pro at all sites in Taiwan, Grason Stadler GSI at BTNRH, and Sony SS-MB150H at UCSF and Johns Hopkins) was located in front of the subject, and the experimental interface was shown on a monitor, inside a sound-proof audiometric booth. Listeners gave their responses by touching a screen or using a mouse.

## Data Analysis

The first analysis compared performance across populations for a given type of pitch inflections. To this aim, performance was averaged over test blocks at a given rate, then translated into a number of hits and a number of false alarms, which could provide an estimate of d′ and beta (i.e. bias in responding)^34^. When performance reached 100% over n trials, it was instead adjusted to 100 × (1 − 1/(2n)), in order to keep d′ within finite values^35^. An analysis of variance (ANOVA) with two between-subjects factors - *hearing status* and *language background* - was performed on the d′ data, separately for each rate that was sufficiently common to NH and CI populations, namely 2, 4, 8, and 16 semitones/sec. Post-hoc comparisons were performed between children in the US and in Taiwan to evaluate the tonal language benefit in each hearing status (Tables 2 and 3). Too few CI subjects could operate at 0.5 and 1 semitone/sec, so differences between NH Taiwanese and NH Americans were assessed by an independent-samples t-test. Similarly, too few NH subjects had been tested on 32 and 64 semitones/sec (as it was too easy for them), and differences between Taiwanese and Americans among users of CI were assessed by an independent-samples t-test.

**Table 2:** Results of the statistical analysis of the d′ data obtained in the labelling task, for isolated sweep rates (shown in the top panels of Figs 1 and 2).

Sweep rate (semitones/sec)	0.5	1	2	4	8	16	32	64
language			F(1,156) = 21.1 *p < 0.001	F(1,160) = 35.3 *p < 0.001	F(1,166) = 49.7 *p < 0.001	F(1,168) = 37.3 *p < 0.001		
hearing			F(1,156) = 49.4 *p < 0.001	F(1,160) = 81.6 *p < 0.001	F(1,166) = 209.3 *p < 0.001	F(1,168) = 157.7 *p < 0.001		
language × hearing			F(1,156) = 12.9 *p < 0.001	F(1,160) = 10.9 *p = 0.001	F(1,166) = 30.6 *p < 0.001	F(1,168) = 19.4 *p < 0.001		
NH-US vs. NH-Taiwan	t(60) = 0.9, p = 0.346	t(93) = 2.0, *p = 0.045	F(1,156) = 41.7 *p < 0.001	F(1,160) = 51.7 *p < 0.001	F(1,166) = 93.3 *p < 0.001	F(1,168) = 64.6 *p < 0.001		
CI-US vs. CI-Taiwan			F(1,156) = 0.4 p = 0.519	F(1,160) = 3.0 p = 0.087	F(1,166) = 1.0 p = 0.321	F(1,168) = 1.3 p = 0.260	t(75) = 4.6, *p < 0.001	t(75) = 3.5, *p = 0.001

**Table 3:** Results of the statistical analysis of the d′ data obtained in the discrimination task, for isolated sweep rates (shown in the bottom panels of Figs 1 and 2).

Sweep rate (semitones/sec)	0.5	1	2	4	8	16	32	64
language			F(1,130) = 8.7 *p = 0.004	F(1,132) = 20.8 *p < 0.001	F(1,135) = 14.1 *p < 0.001	F(1,137) = 25.7 *p < 0.001		
hearing			F(1,130) = 49.7 *p < 0.001	F(1,132) = 111.0 *p < 0.001	F(1,135) = 163.7 *p < 0.001	F(1,137) = 138.9 *p < 0.001		
language × hearing			F(1,130) = 6.5 *p = 0.012	F(1,132) = 6.6 *p = 0.011	F(1,135) = 9.7 *p = 0.002	F(1,137) = 7.8 *p = 0.006		
NH-US vs. NH-Taiwan	t(52) = 0.5, p = 0.654	t(81) = 1.3, p = 0.194	F(1,130) = 22.2 *p < 0.001	F(1,132) = 36.8 *p < 0.001	F(1,135) = 33.7 *p < 0.001	F(1,137) = 43.8 *p < 0.001		
CI-US vs. CI-Taiwan			F(1,130) < 0.1 p = 0.808	F(1,132) = 1.5 p = 0.223	F(1,135) = 0.2 p = 0.696	F(1,137) = 2.0 p = 0.161	t(57) = 0.4, p = 0.660	t(57) = 0.7, p = 0.507

The second analysis was focused on comparing the rate of pitch inflections that was required for the different populations to reach a fixed level of discriminability, namely d′ = 0.77. This normative value was chosen to ease comparisons with studies using adaptive staircases. To this aim, performance data were fitted (separately for up-sweeps and down-sweeps) with a Weibull function, using the maximum-likelihood technique^36,37^. The scale of sweep rates varied logarithmically (base 2). The fitting procedure was facilitated by Gaussian priors. The lower and upper bounds were probed around performance at, respectively, the shallowest and steepest rate available, and a standard deviation of 30%. There was no prior for the position of the inflection point but its slope was searched around a mean of 1 and a standard deviation of 3. Subsequently, d′ fits were generated from the fits of up- and down-sweeps, enabling extraction of a threshold (expressed in semitones/sec) at exactly d′ of 0.77 for each child. In this process, the performance data could reached 100% but the Weibull fit was limited at 100 × (1 − 1/(2n)) to keep the resulting d′ fit within finite values^35^. In a number of cases (18% of Americans with CI, 18% of NH Americans, 11% of Taiwanese with CI, and 1% NH Taiwanese), the maximum likelihood technique could not find any acceptable Weibull fit to the performance of down-sweeps because it exhibited a flat or non-monotonic pattern, an issue made transparent in our earlier study^32^. Occasionally, this also occurred for up-sweeps (8% of Americans with CI, 6% of Taiwanese with CI, 2% of NH Americans). Whenever this happened, the data were fitted with a straight line corresponding to the average performance across all rates tested. In those cases, the d′ fit was thus primarily based on the Weibull fit obtained from the other sweep direction. Finally, thresholds were assigned to “chance” (and excluded from further analysis) whenever the d′ fit could not exceed 0.77 by a sweep rate of 256 semitones/sec. To evaluate the measurable thresholds statistically, an ANOVA with two between-subjects factors - *hearing status* and *language background* - was performed, and post-hoc comparisons between children in the US and Taiwan tested the tonal language benefit in each hearing status (Table 4).

**Table 4:** Results of the statistical analysis of the thresholds extracted at d′ = 0.77 (shown in Figs 4 and 5).

Task	1I-2AFC	3I-2AFC
language	F(1,148) = 41.6 *p < 0.001	F(1,118) = 5.9 *p = 0.017
hearing	F(1,148) = 143.0 *p < 0.001	F(1,118) = 151.3 *p < 0.001
language × hearing	F(1,148) = 8.3 *p = 0.005	F(1,118) = 11.5 *p = 0.001
NH-US vs. NH-Taiwan	F(1,148) = 53.2 *p < 0.001	F(1,118) = 26.6 *p < 0.001
CI-US vs. CI-Taiwan	F(1,148) = 5.4 *p = 0.021	F(1,118) = 0.3 p = 0.558

## Results

### Analysis of d′

**Figure 1:**
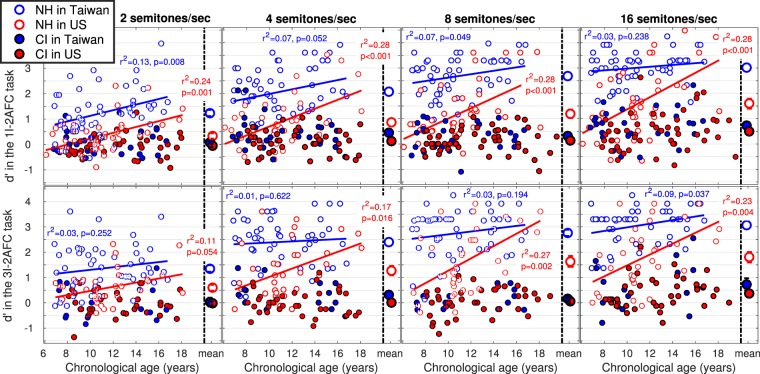
d′ data across children for 300-ms sweeps of ±2 (most-left) ±4 (middle-left), ±8 (middle-right), and ±16 (most-right) semitones/sec, in two tasks where the child was asked to label the direction of a single sweep (top panels) or discriminate between sweeps of opposite direction (bottom panels). Means are on the right-hand side of each panel, and error bars represent one standard error. A higher d′ reflects a more acute sensitivity. At these rates, none of the regressions reached significance among children with CIs, and age effects were stronger for NH children in the US than in Taiwan.

Figure 1 shows the d′ data measured for each of the four populations in the labelling task (top panels) and the discrimination task (bottom panels). In both tasks, the results of the ANOVAs reveal significant main effects and interaction (Tables 2 and 3). The large deficits exhibited by children with CI were expected, and therefore interactions were explored for the effect of language background. Post-hoc tests revealed that NH Taiwanese outperformed NH Americans at 2, 4, 8, and 16 semitones/sec, and consistently in both tasks. This represents strong evidence for a tonal language benefit among NH children, with an effect size from 1.0 to 1.5 gain in d′. In contrast, there were too few differences between Taiwanese and Americans wearing CI at those rates.

**Figure 2:**
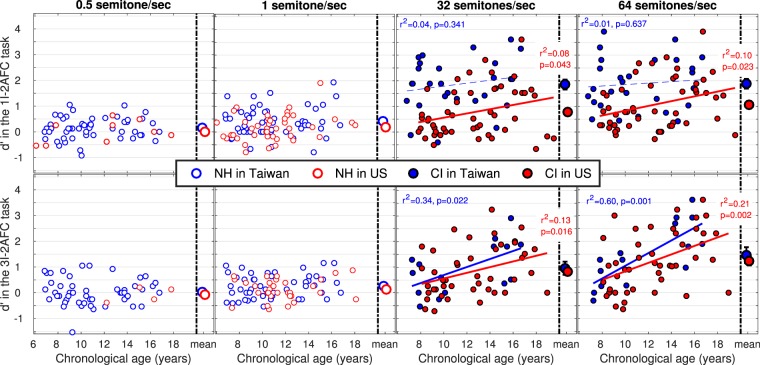
d′ data across children for 300-ms sweeps of ±0.5 (most-left) ±1 (middle-left), ±32 (middle-right), and ±64 (most-right) semitones/sec, in the labelling (top) or discrimination (bottom) task. Means are on the right-hand side of each panel, and error bars represent one standard error. At the shallow rates, none of the correlations reached significance among NH children, and at steep rates, age effects were more consistent for implanted children in the US than in Taiwan.

Figure 2 shows the d′ data measured for the two NH populations at very shallow rates (left panels) and for the two CI populations at very steep rates (right panels). As illustrated on the bottom panels, there was no tonal language benefit among NH children or among children with CI in the discrimination task (stats reported in Table 3). However, in the labelling task (top panels, with stats reported in Table 2), there was a tonal language benefit among children with CI at both 32 and 64 semitones/sec, and a small benefit among NH children at 1 semitone/sec (although it would not survive Bonferroni correction).

Linear regressions investigating the effects of chronological age revealed a striking asymmetry. For NH children in Taiwan, regression coefficients were not all significant between 2 and 16 semitones/sec (Fig. 1) and never explained more than 13% of the variance. In contrast, for NH children in the US, the effect of age was stronger: regression coefficients were consistent between 2 and 16 semitones/sec and explained up to 28% of the variance. This asymmetry was paralleled in the CI groups: at 32 and 64 semitones/sec, regression coefficients were significant for children in the US in both tasks but only in the discrimination task for children in Taiwan.

### Analysis of thresholds

**Figure 3:**
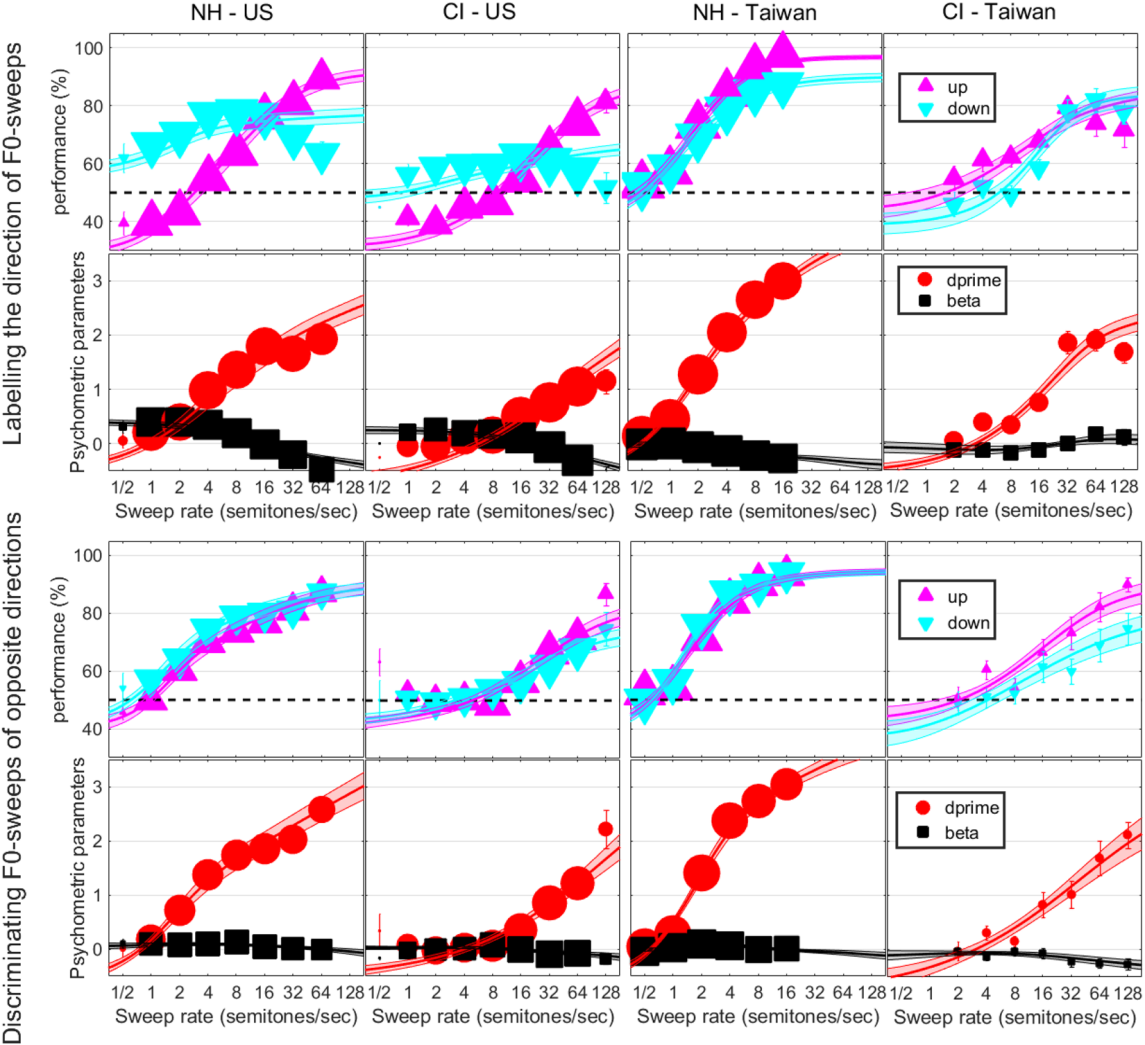
Psychometric functions expressed as percent correct for up- and down-sweeps and subsequently converted into d′ values, in the 1I-2AFC labelling task (top) and the 3I-2AFC discrimination task (bottom), as a function of the rate of F0-sweeps expressed in semitones per second. Symbols represent the weighted mean and error bars indicate one weighted standard error of the mean. Note that the size of symbols relates to the weights on each condition reflecting the number of trials collected across all subjects. Lines and surfaces are the Weibull fits with one standard error.

Figure 3 shows performance (in % correct) for up- and down-sweeps in each population and each task, along with the corresponding psychometric parameters, d′ and beta. This illustrates more clearly how d′ improved with increasing sweep rates. In the discrimination task, performance started around 50% for shallow rates and increased monotonically as sweeps became steeper, with little bias with regard to the direction. This was not the case in the labelling task, in which performance differed from chance level at shallow rates. In the US more particularly, children were biased to respond “down” more often at shallow rates, and “up” more often at steep rates. This anomaly has been extensively discussed^32^ and reflects that some listeners (here children, but this also applies to adults) judge a given sweep by comparison with many sweeps presented earlier. When sweeps hit distinct F0 ranges depending on their rate (which has to occur for very steep sweeps), the percept of F0 height becomes difficult to ignore, and in this respect Taiwanese children (both NH and CI) seem more immune than American children (see general discussion). Note that this bias can cause non-monotonicity of the psychometric function, which the Weibull fit cannot follow. Nonetheless, when combining both directions, the d′ fits are in relatively good correspondence with the d′ data, such that the threshold derived for a given child was already a good representation of his/her sensitivity.

**Figure 4:**
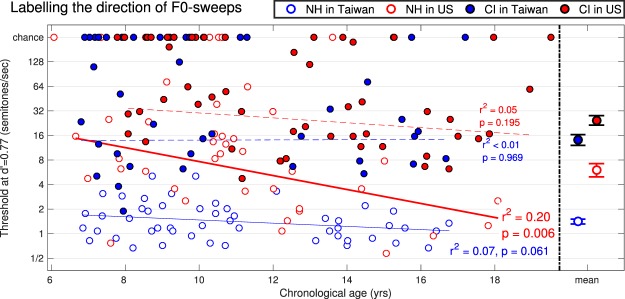
Individual thresholds across children and means on the right-hand side with one standard error, for the labelling task. Here, a better sensitivity is reflected by a lower threshold. Children who could not perform at this level of d′ are reported as chance on the top-end of each panel.

**Figure 5:**
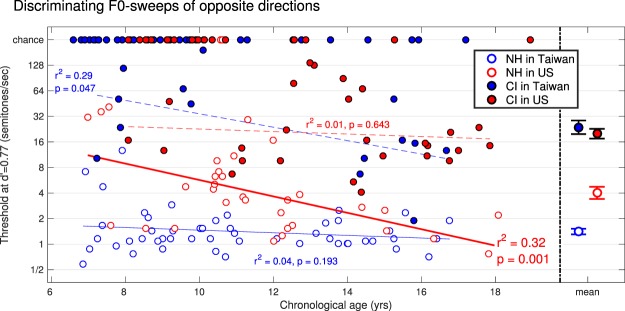
Same as Fig. 4 for the discrimination task.

Figures 4 and 5 show the thresholds, i.e. the values of sweep rate that would be required for each child to reach a common level of discriminability namely d′ of 0.77. Note that there were some children who did not obtain 80% performance during practice and there were children who passed the criterion but provided data which were very close to chance such that a threshold was not measurable at d′ = 0.77. All these subjects are shown here as “chance” on the top-end of each panel. It is clear that many children with CIs (of both language backgrounds) could not do these tests. Even with extremely steep sweeps of 128 semitones/sec (corresponding to a change of 3.2 octaves within 300 ms), many of them, especially the younger children, could not tell whether the pitch was rising or falling, and discriminate between the two. This speaks to the considerable limitations in complex pitch coding currently offered by envelope-based devices. Among all participants who provided a measurable threshold, the statistical results (reported in Table 4) revealed consistent findings in both tasks: a main effect of language background, a main effect of hearing status, and an interaction. Delving further into the interaction, NH Taiwanese outperformed NH Americans in both tasks, and Taiwanese users of CI outperformed American users of CI in the labelling task only. In other words, this is qualitatively the same pattern that emerged from the first analysis.

The effect of chronological age was again evaluated with linear regressions. This factor accounted for 20% and 32% of the variance among NH Americans while it did not reach significance for NH Taiwanese. Among children with CIs, many could not perform the tasks, so age effects were inconsistent. Overall, this is also similar to the pattern that emerged from the first analysis.

Given the different slopes of age effects between the two NH populations, an additional analysis was performed to evaluate the effect of age on the tonal language *benefit*. To this aim, the age axis was split into 22 bins with a width of 2 years, equally spaced every 0.5 year. The thresholds for the children falling into a given bin were pooled together, and a mean and standard deviation was calculated. By computing the difference between the means of the two NH populations divided by their pooled standard deviation, an estimate of effect size was derived in each bin. A linear trend could well account for the progressive reduction in the tonal language benefit with chronological age (r^2^ = 0.51, p < 0.001 in the labelling task; r^2^ = 0.23, p = 0.024 in the discrimination task) which would have completely disappeared by age 20 and 22, respectively in each task.

### RT data

For each trial, RT was stored separately for correct and incorrect responses. In each population and each task, averaged RT for correct responses decreased as sweep rate increased. The effect was best described by linear regressions, decreasing by several hundred milliseconds (decreasing by 100 ms at minimum and 600 ms at most) from the shallowest to the steepest rates. This pattern was largely expected and can be taken as evidence for diligent behavior^38^: children presumably tried to perform as well as possible because they took a bit more time to provide their responses when facing subtle cues.

### Probing other factors

Thresholds obtained by children with CI were also examined as a function of age at implantation and duration of CI experience, but none of the correlations were significant, even when pooling the two populations together (p > 0.204). Among other factors of interest, there were no differences between unilaterally and bilaterally implanted children in either task [F(1,59) = 2.3, p = 0.133; F(1,41) = 3.0, p = 0.089], no effect of the side of ear tested [F(1,59) = 0.1, p = 0.722; F(1,41) = 2.5, p = 0.120], and no interaction between the two [F(1,59) < 0.1, p = 0.858; F(1,41) = 3.6, p = 0.064]. The effect of contralateral hearing aid use did not reach significance [F(1,61) = 3.3, p = 0.072; F(1,43) = 0.4, p = 0.538], and there was no difference between devices built by different manufacturers [F(2,60) = 0.3, p = 0.768; F(2,42) < 0.1, p = 0.962].

## General Discussion

A clear tonal language benefit was observed among NH children. This was true for the ability to label the direction of a single sweep, as well as the ability to discriminate between sweeps of opposite direction. This was demonstrated first by the finding that NH Taiwanese obtained higher d′ than their American peers across a very large diversity of sweeps, from F0s that rose/fell by only 60 cents to as much as 4.8 semitones within 300 ms (Fig. 1). Second, this was demonstrated by the finding that NH Taiwanese needed sweep rates of only 1.4 semitone/sec whereas their American peers needed sweep rates of 5.4 semitone/sec (on average across tasks) to achieve the same value of d′ (Figs 4 and 5). Perhaps even more interesting is the fact that this tonal language benefit would not survive in adulthood. Indeed, chronological age had a stronger role for Americans than for Taiwanese. Presumably, Mandarin-speaking children learn early on in life that pitch inflections are critical to pay attention to, and this gives them an edge over young English-speaking children in dynamic-pitch tasks. Therefore, researchers should probe the early years of language development when seeking evidence for tonal language benefits.

What is also striking is that this tonal language benefit was absent in our previous study^30^ measuring static F0 sensitivity, using otherwise similar design, equipment, and analysis. This means that the tonal language benefit may not generalize to pitch perception overall, at least in children. Rather, it seems to concern the sensitivity to continuous changes in pitch over time (as in speech) and not discrete ones (as in a melodic sequence of piano notes), providing no support for cross-domain generalization (speech to music). This finding also supports the general conclusion based on the literature that if tonal language benefits are to be found in pitch perception tasks, they are more likely present in tasks that are closely related to the demands of the tonal language.

The tonal language benefit is often interpreted in terms of enhancement in the internal representation of F0, largely owing to the number of studies that showed a stronger encoding of periodicity in brainstem FFRs of tonal language speakers (see section A of the introduction). The NH data in this study are in line with this standard interpretation. The lack of tonal language benefit among CI users in the discrimination task is also consistent with this framework, since F0 representation is severely degraded within this population. As such, CI users (regardless of their language background) must derive a pitch percept from a cue that is poorly coded in terms of spectral harmonic structure and in terms of temporal waveform^24^. There is much interest in understanding what exactly that cue is, whether it is primarily derived from temporal envelope periodicity^23,39–43^, or a rough spectral centroid constructed from place cues^44–46^, and whether different pitch percepts can be interchangeable^47,48^. One result, however, that achieves consensus in the field is that the brains of CI users can adapt to the signal degradations in the input to some extent. There is a plethora of clinical evidence for the benefits of early implantation^49–58^. Unfortunately, much of this clinical evidence is focused on speech and oral communication, and it is doubtful that neural plasticity in the early stages of development can overcome the limitations of CIs in terms of pitch. Both the present and previous results^30^ cast a pessimistic light in this regard: neither age at implantation nor years of CI use had any impact on the sensitivity to static or dynamic F0, which remained overall poor. Here, thresholds were about 16–32 semitones/sec (on average) and this effect size is actually underestimated because it is based on the children who could provide a measurable threshold at a d′ of 0.77. Even with sweeps of 128 semitones/sec, 34% of children with CIs could not tell whether the pitch was rising or falling, and 44% could not discriminate between the two. Yet, most of these children were implanted before 4–5 years of age and had at least six years of experience with the device. Thus, we believe that it is rather unlikely that neuroplasticity will suffice to recover a sharp internal representation of F0 in children wearing cochlear implants. Note that this is not the first study to reach this conclusion: although there is a quick adaptation following device activation, children seem to suffer largely from the same difficulties as adults (wearing cochlear implants) in pitch-related tasks such as emotion recognition, prosody perception/production, and lexical tone identification^28,59–67^.

From this standpoint, the discovery that Taiwanese children with CIs outperformed their US counterparts only in the labelling task is of great interest. This implies that there is an additional explanation for this phenomenon, potentially unrelated to the internal representation of F0 and specific to what happens in the brain during “labelling”. One particularity of the labelling task (discussed in depth^32^) is that a single sound is presented without explicit reference to a common pitch range. When a sweep sounds roughly flat, the listener may respond based on the pitch range, i.e. “going up” for a high-pitch range and “going down” for a low-pitch range. That is, listeners may confuse the meaning of pitch direction with that of pitch height. This is not a strategy that is only recruited by CI users; NH listeners fall back on it too, provided that the sweeps are sufficiently shallow to be perceived as flat. However, the problem is somewhat exacerbated for CI users because they cannot perform the task until the sweeps are very steep. Sweep rates of 64 or 128 semitones/sec necessarily elicited a high pitch range (either from the beginning or the end of the sound). Since the sweeps were only 300-ms long, children with CI could perceive them as one diffuse high range without any motion to them, which contrasted with the relatively low range used for other (shallower) sweeps. In other words, in the 1I-2AFC, listeners accumulate precedent trials as internal references which bias the decision made on a given trial. This problem does not arise in the 3I-2AFC task because this reference is made explicit by having the three sweeps covering the same pitch range (no bias between up/down in the bottom panels of Fig. 3). Therefore, we speculate that the advantage exhibited by Taiwanese over American children wearing CIs in the labelling task is in fact a conceptual advantage, derived from a greater familiarity with the task demands. Through their natural and intensive exposure to Mandarin, the Taiwanese children may have disambiguated the notions of pitch height and pitch direction better than American children, rendering them more immune to the bias of the height of the pitch range covering steep sweeps. In fact, from multidimensional scaling studies across languages, there is already some evidence that speakers of tonal languages weigh the direction dimension more heavily than English speakers, whereas English speakers weigh the height dimension more heavily than tonal language speakers^68^, and this has repercussions in the strategies that people use when learning a new language. For example, English speakers learnt to categorize Cantonese tones by relying heavily on their height whereas Mandarin speakers learnt to categorize them by relying more on their direction^69^. Even though this study examined young adults, none of them had any familiarity with Cantonese, so it bears some similarity to children acquiring their native language. This supports a different interpretation of the tonal language benefit, namely that children pay particular attention to the pitch dimension that is most phonetically relevant in their language, which in this study favored the Taiwanese whereas it favored the Americans in our previous study^30^. This difference alone in dimension-weighing may be sufficient in accounting for the diametrically opposite pattern of results in our two studies.

## Summary

We measured the full psychometric functions in four groups of children (Taiwanese and Americans, with normal hearing or wearing cochlear implants) for the sensitivity to linear glides in complex pitch (broadband harmonic complexes with rising/falling F0). NH Taiwanese outperformed NH Americans across a very large range of sweep rates, and for both tasks (discrimination and labelling), consistent with the idea that speaking a tonal language sharpens the fine-grained internal representation of F0. The largest differences between these populations occurred for the youngest children: NH Taiwanese behaved relatively adult-like at 6 years of age whereas NH Americans progressively caught up with NH Taiwanese up to 18 years of age, presumably compensating their lack of tonal exposure by cognitive skills (more advanced than those of the 6 year olds NH Americans). Given the current slope of regression with chronological age, this tonal language benefit would not be observed in adulthood.

As expected, implanted children struggled in the pitch tasks. Impressively, however, the tonal language benefit transferred to these populations in the labelling task only. This observation opens up a novel account for the tonal language advantage phenomenon that is potentially unrelated to the quality of the internal representation of F0. We speculate that this advantage might partly reflect the familiarity of encountering pitch in a dynamic context, where Taiwanese children avoided confusions with other meanings (e.g. pitch height). Despite stringent limitations in F0 coding, growing up in a tonal language environment could help children in reaching a decision with shallow (for NH) or degraded (for CI) pitch contours.

## Data Availability

All data, materials, and analysis codes are publicly available on the Open Science Framework link: https://osf.io/7pkjq/?view_only=bc343b70162f475a84f135bd2bca8dcf.
